# Adherence Is More Than Just Being Present: Example of a Lay-Led Home-Based Programme with Physical Exercise, Nutritional Improvement and Social Support, in Prefrail and Frail Community-Dwelling Older Adults

**DOI:** 10.3390/ijerph18084192

**Published:** 2021-04-15

**Authors:** Christian Lackinger, Igor Grabovac, Sandra Haider, Ali Kapan, Eva Winzer, K. Viktoria Stein, Thomas E. Dorner

**Affiliations:** 1 Karl-Landsteiner Institute for Health Promotion Research, 3454 Sitzenberg-Reidling, Austria; KatharinaViktoria.Stein@bvaeb.at (K.V.S.); thomas.dorner@bvaeb.at (T.E.D.); 2 Social Insurance Fund for Public Service, Railway and Mining Industries, Gesundheitseinrichtung Sitzenberg-Reidling, 3454 Sitzenberg-Reidling, Austria; 3 Department of Social and Preventive Medicine, Centre for Public Health, Medical University of Vienna, 1080 Vienna, Austria; igor.grabovac@meduniwien.ac.at (I.G.); sandra.a.haider@meduniwien.ac.at (S.H.); ali.kapan@meduniwien.ac.at (A.K.); eva.winzer@meduniwien.ac.at (E.W.)

**Keywords:** complex intervention study, adherence, lay-led intervention, community-dwelling people, frailty, buddy

## Abstract

Background: Little is known about the implementation of lifestyle interventions in frail, community-dwelling people. This study highlights different domains of adherence to explain an effectively delivered home-based intervention. Methods: Eighty prefrail and frail persons (≥65 years) participated in a physical training, nutritional, and social support intervention over 24 weeks. A detailed log book was kept for comprehensive documentation in order to assess adherence and further organizational, exercise, and nutritional parameters. Results: Participants reached an adherence rate (performed home visits/number of planned visits) of 84.0/80.5% from week 1–12/13–24. Out of those, 59% carried out ≥75% of the offered visits. Older age was associated with a higher adherence rate. A mean of 1.5 (0.6) visits/week (2 were planned) were realized lasting for a mean of 1.5 (0.9) hours (154% of the planned duration). Per visit, 1.2 (0.6) circuits of strength training were performed (60.5% of the planned value) and 0.5 (0.3) nutritional interventions (47%). After twelve months, 4.2% still carried out the home visits regularly and 25.0% occasionally. Conclusion: Adherence is much more than “being there”. Adherence rate and category are limited parameters to describe the implementation of a complex lifestyle intervention, therefore a comprehensive documentation is needed.

## 1. Introduction

Due to demographic changes, the global population is getting older. In such an aging population, frailty is fast becoming an important challenge for health-care systems [1]. Sarcopenia, low physical activity levels, reduced muscle strength, exhaustion, malnutrition, depression, low social support, impaired cognitive function, and chronic inflammation are parameters that contribute to the frailty syndrome [2,3,4,5]. According to the Survey of Health, Aging and Retirement in Europe (SHARE), in the European population aged 50+, 14.7% and 6.9% of community-dwelling people were found to be prefrail or frail, respectively [6]. It is estimated that in Austria by the year 2050, approximately 356,000 people will be frail and 1.5 million prefrail in a total population of 9 million inhabitants [7].

To prevent older persons from becoming frail or to improve the personal health resources in frail people, different lifestyle interventions have been developed [8,9,10]. Through such lifestyle interventions, community-dwelling frail persons can be supported to improve different health aspects and continue an autonomous life at home. Besides physical activity and nutritional aspects, social interaction and support—especially improving the regular contact with other people—seem to be key factors for sustainable implementation of lifestyle programs [11]. Community-dwelling older adults often suffer from social isolation and have low participation in communal activities, so home-based programs that enable social interaction with others should help reach a high level of adherence. Many older people fail to adhere to lifestyle recommendations not because they purposefully decide to do so, but due to a lack of social support, missing coping skills, acute illnesses, or cognitive decline that hinder the sustainable implementation of healthy lifestyle changes [12]. Lifestyle interventions, that are exclusively focused on health outcomes and do not provide opportunities for social interaction, might be limited in obtaining long-term effects [13,14].

Most research of lifestyle interventions in prefrail and frail populations were carried out by health professionals. In addition to the work of health professionals, the support of lay persons in medical care and lifestyle interventions is becoming more and more important [15]. In other sectors, the involvement of lay persons has resulted in the successful delivery of lifestyle interventions and has led to acceptable adherence [16].

Adherence to an intervention is commonly defined as the actual participation level compared to planned sessions or visits [17]. Thus, the adherence rate can be defined as the number of realized sessions relative to the number of offered ones [18]. Within frail persons, adherence rates between 55% and 96% have been reported for lifestyle programs [18,19,20]. Due to the fact that there are some participants who achieve a good adherence rate while others do not, the differentiation between adherence categories contains important information about the number of adherent participants in a lifestyle program. An attendance of ≥75% of all offered interventions is described as good/acceptable adherence in many studies [21,22]. Spink et al. reported that 52% of a frail study population completed ≥75% of a home-based exercise program [23]. In many lifestyle-intervention studies, a certain number of drop-outs is inevitable. Therefore, to interpret adherence, the drop-out rate also has to be considered. For instance, Barker et al. reported that 95% of the participants that completed the study intervention attended ≥ 75% of the offered classes [24]. Within this calculation, 23% of the participants who dropped out were not included. The assessment of adherence differs between studies: while some studies used a questionnaire [23], others used log books or diaries [20]. Consequently, prospectively assessed adherence cannot be accurately compared to retrospective data without limitations, because of the recall bias. Furthermore, retrospectively self-reported data might overestimate adherence [25].

In addition to assessing adherence rates and categories, a detailed documentation of the delivered measures (as opposed to those offered) brings further information in complex intervention studies. The nature of many clinical, health promotion, and public health interventions is complex, which may be described by using some key parameters: (1) the number of interactive components; (2) the number of different people that deliver an intervention; (3) the number of organizations that are targeted or involved in the intervention; (4) the variability of the outcomes and the flexibility or tailoring of the intervention [26,27]. Without reporting how an intervention was delivered, randomized controlled trials that are focused exclusively on outcomes might lose validity [28]. A comprehensive documentation of the actually implemented intervention adds important information to outcomes, because it helps clarifying the question why an intervention works and how it was delivered. Thus, the impact of outcome-driven public health investigations is improved through a comprehensive documentation because the fidelity to the study protocol, the level of the delivered intervention, and the quality of the implementation can be determined [29]. To illustrate further, in a lifestyle study with overweight and obese patients desired effects were measured, although less than 20% of the participants reached high adherence levels [30]. Differences in implementation of the intervention compared to the study protocol were evident. Although the interventions were offered as planned, only parts were realized by the participants, and still positive effects were reported. Thus, we hypothesize that there is a gap between planned lifestyle-interventions and the practical implementation. Due to the fact that planning lifestyle-interventions are based on physiological-outcome-driven research, the measures might not consider prerequisites for long-term feasibility in a real-world setting. The disregard of these circumstances promotes deviations of the performed intervention compared to the study protocol. In contrast to physiological-outcome-orientated studies, lifestyle studies are not exclusively focused on the maximization of an effect in a defined period of time, they aim to implement effective habits in the long run.

To the best of our knowledge, limited information was published so far regarding the prospectively assessed adherence rate completed with a comprehensive documentation regarding the quality of the performed interventions in home-based lifestyle programs in prefrail/frail community-dwelling older adults that were carried out by lay persons. This current study was part of a randomized, controlled trial, where lifestyle interventions (social support, physical activity, and nutritional aspects) were delivered by lay volunteers to community-dwelling prefrail/frail older adults and aimed to assess how a complex lifestyle intervention was actually performed in comparison to what was planned. Adherence and parameters of the comprehensive documentation were assessed over 24 weeks [31].

It was the aim of this analysis to investigate different domains of adherence to a physical training, nutrition, and social support intervention in a special setting (community-dwelling prefrail/frail persons). Furthermore, it was the aim to examine factors associated with the adherence rate.

## 2. Materials and Methods

### 2.1. Study Design

This randomized, controlled trial took place in Vienna (Austria) and included a physical training and nutrition (PTN) group and a social support (SoSu) group. The study design and planned interventions were published before the participants were enrolled [31]. The sample size calculation was described in detail in the study protocol and was based on handgrip strength. Participants in both groups were community-dwelling prefrail and frail persons (aged > 65 years), who were visited at home from lay volunteers called “buddies” over 24 weeks. In this face-to-face intervention, home visits were planned twice a week and each session was scheduled for 1 h. Eighty couples of prefrail/frail persons and their buddies participated in the study. A buddy was allowed to support more than one prefrail/frail participant. The health status, quality of life, nutritional status, and physical activity parameters were already published earlier [32,33,34].

#### 2.1.1. Characteristics of the Participants

Prefrail and frail community-dwelling people older than 65 years were enrolled in this study. The frailty status was obtained using the Frailty Instrument for Primary Care of the Survey of Health, Ageing, and Retirement in Europe (SHARE-FI) [35]. For the case that a robust person was undernourished, participation in the study was accepted. Malnutrition was assessed with the Mini Nutritional Assessment Short-Form (MNA-SF) questionnaire [36]. People with planned admission to a nursing home, with acute or planned chemo- or radiotherapy, with impaired cognitive functions, with insulin-treated type 2 diabetes mellitus, or chronic obstructive pulmonary disease stage III or IV were excluded from the study.

The prefrail/frail participants were visited by lay volunteers (buddies) who were aged ≥50 years [37]. Both, the prefrail/frail participants and their buddies were asked to participate in the study in either the PTN or SoSu group for at least six months.

#### 2.1.2. Intervention: Week 1–12

The intervention was divided into two phases, with each period lasting for 12 weeks. Thus, over a period of 24 weeks, home visits were planned twice a week. During the first 12 weeks, in the PTN group, buddies and the prefrail/frail subjects performed a standardized strength training twice a week, for which the buddies were trained. The buddies also addressed various nutritional topics, e.g., hydration in a standardized manner. The education of the buddies was delivered by the project staff, which included medical doctors, nutritional scientists, nutritionists, sport scientists, physiotherapists, and psychologists. The education consisted of four modules, each lasting for 3 h. In short, the standardized physical activity intervention consisted of 5 min of mobilization and warm-up exercises and 30 min of strength training. According to the study protocol, two circuits with six different strength exercises, each with 15 repetitions, were planned within a visit. The standardized nutritional intervention was based on three main messages: fluid intake, animal and plant protein intake, and appropriate energy intake. The other component of the nutritional intervention was the use of a modified Harvard Healthy Eating Plate [38], the so-called “Healthy for Life Plate” aimed to exemplify the quantity and composition of daily food intake [31].

Over the first 12 weeks, for the SoSu group, the buddies were encouraged to visit the older persons twice a week and performed various social activities together, such as playing games, talking, sharing interests, or doing cognitive training exercises. Within this period, the prefrail/frail participants in the SoSu group did not perform the exercise regime, nor was the nutritional support given.

#### 2.1.3. Intervention: Week 13–24

The participants in the PTN group continued the combined physical activity and nutritional intervention regime for further 12 weeks. Consequently, the PTN group received 24 weeks of physical activity and nutritional intervention.

From week 13–24, the SoSu group also received the combined physical training and nutritional intervention, which was equal to the action that took place in the PTN group.

### 2.2. Measurements of Different Aspects of Adherence

**Figure 1:**
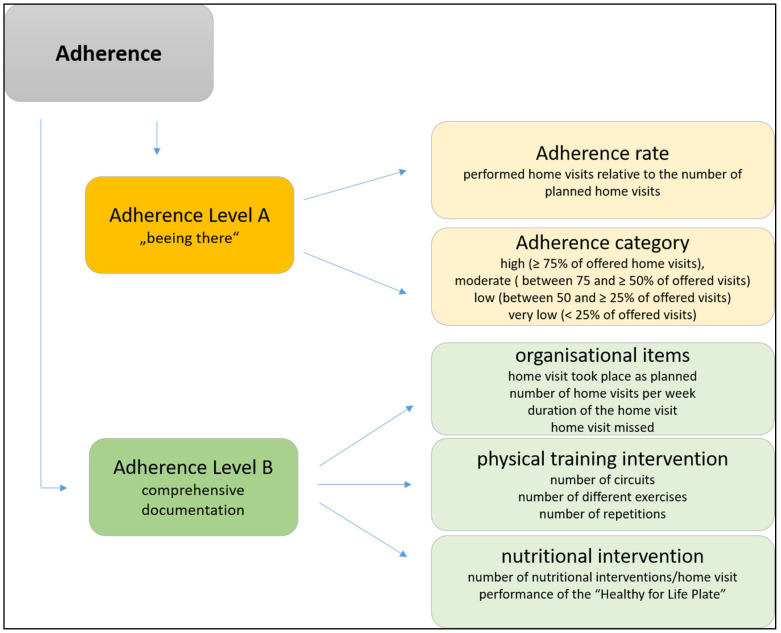
Different domains of adherence.

For 24 weeks for each single visit a comprehensive documentation was done by the buddies using a standardized log book. This included organizational items, but also details of the physical training and nutritional intervention. The different domains of adherence were assessed after 12 weeks (follow-up 1) and after 24 weeks (follow-up 2) and are shown in Figure 1. After 12 months, the long-term participation (continuation) was assessed.

#### 2.2.1. Log Book Determined Adherence Rate and Category

Based on the log book data, the adherence rate, defined as the percentage of performed home visits relative to the number of planned home visits was calculated [18,39].

This was later further subdivided into (adherence category):

- high (carried out ≥75% of offered home visits),
- moderate (carried out between 75 and ≥50% of offered home visits),
- low (carried out between 50 and ≥25% of offered home visits), or
- very low (drop-outs or less than 25% of offered home visits) [21].

Participants who discontinued participation because of medical or personal reasons, or who died, were designated as drop-outs [31]. To define retention, the number of participants who completed the follow-up investigations divided by the number of participants who completed the baseline investigation was used [39].

#### 2.2.2. Comprehensive Documentation of the Lifestyle Intervention

In addition to the adherence rate and category, further details of the nutritional education and physical training were documented to quantify the efficiency of the delivered intervention. These data included:

- organizational items: home visit took place as planned (yes/no); number of home visits per week; duration of the home visit; home visit missed due to the prefrail/frail participant or the buddy;
- physical training intervention: number of circuits; number of different exercises; number of repetitions;
- nutritional intervention: number of nutritional interventions per home visit (≥1 nutritional message discussed); performance of the “Healthy for Life Plate” during a visit (yes/no).

The quality of the actually delivered intervention was calculated as the percentage of the planned intervention according to the study protocol [31].

#### 2.2.3. Self-Perceived Adherence Rate

After the intervention week 24, all participants (prefrail/frail persons and their buddies) retrospectively answered three questions regarding self-perceived adherence to the program over the last 12 weeks. The questions were:

- “How many home visits were carried out?”
- “How many times were nutritional aspects discussed?”
- “How many times was the predetermined physical activity program realized?”

#### 2.2.4. Continuation

The long-term maintenance of the visits six months after the official ending of the study was defined as continuation. Therefore, telephone interviews were performed 12 months after the intervention started. Buddies were asked:

- “Did you regularly continue the study intervention (home visits with physical training and nutritional intervention) in the last 6 months?” (yes/no)
- “Did you sporadically continue the study intervention in the last 6 months?” (yes/no)
- “Did you stop the home visits in the last 6 months?” (yes/no)
- “How many home visits were performed in the last 6 months?” (number)
- “How many physical training interventions were performed in the last 6 months?” (number)
- “How many nutritional interventions were performed in the last 6 months?” (number)

#### 2.2.5. Association with Adherence Rate

Participants’ characteristics (age, sex, education level, and SHARE-FI score) were analyzed to assess possible associations with the adherence rate.

### 2.3. Ethical Considerations

The study was approved by the local ethics committee (Ref: 1416/2013) and was registered at ClinicalTrials.gov (NCT01991639). The study was performed in accordance with the Declaration of Helsinki [40].

### 2.4. Statistical Analyses

To explore the adherence, descriptive statistical analyses were used. The baseline characteristics of the prefrail/frail participants and buddies are given by frequencies or percentages. *t*-tests and chi-square tests were used to assess differences in adherence.

In addition, chi-square tests were used to test the baseline variables associated with adherence. From all the prefrail/frail participants, two groups were generated: participants that accumulated ≥75% of all planned visits over the last 12 weeks were defined as the high-adherence group, while those who realized <75% were defined as the low- to moderate-adherence group. All participants (PTN and SoSu) were analyzed together at follow-up 2. After the median split, the variable age was dichotomized for the prefrail/frail older persons and for the buddies. Prefrail/frail participants were divided into over 81 and ≤81 years of age; buddies were divided into over or ≤59 years.

## 3. Results

### 3.1. Baseline Characteristics of the Participants

In this study, 80 couples were included. Baseline data for 80 prefrail/frail persons and their buddies (N = 70) were investigated. According to the SHARE-FI classification almost all participants were prefrail or frail. Further baseline characteristics of the study population are shown in Table 1.

### 3.2. Adherence Rate & Category

As shown in Table 2, adherence data were calculated for the PTN and SoSu groups at both follow-up points. Although the data do show a higher adherence rate in the PTN group compared to the SoSu group after 12 and 24 weeks, this was not statistically significant in the between-group comparisons. According to the comparison within the same group adherence declined within the second 12 weeks-period, but again not significantly. Adherence categories were significantly different in the PTN group between follow-ups (*p* = 0.018). Adherence is presented in Table 2.

After 24 weeks, 21 prefrail/frail participants (26%) had dropped out. Of these drop-outs, three (14%) died, another six (28%) dropped out for medical reasons and 1 (5%) person showed an aggressive attitude towards the buddy and was therefore excluded. The majority (11 persons, 52%) dropped out for unspecified personal reasons.

### 3.3. Quality of the Intervention (Based on the Comprehensive Documentation)

After 12 weeks, the number of missed visits (3.0 ± 3.8) in the SoSu group due to buddies was significantly higher than in the PTN group (1.6 ± 2.2 visits, *p* = 0.028). In addition, at follow-up 2, significantly more visits were cancelled by the prefrail/frail individuals than by the buddies. After 24 weeks, 59 prefrail/frail participants were included in the analysis based on the completed log book. The quality of the delivered interventions is shown in Table 3. Only couples that performed the PTN regime in the observed period of time were included in the analysis.

### 3.4. Variables Associated with Adherence Category

The SHARE-FI category and age were significantly associated with the adherence category in the prefrail/frail participants (Table 4), while no such association was found for the buddies (Table 5).

### 3.5. Self-Perceived Adherence Parameters

The prefrail/frail participants reported that a mean of 20.7 (4.9) of 24 possible home visits were carried out, which is in accordance with an adherence rate of 86.1%. According to the prefrail/frail persons, the number of realized physical activity interventions was 19.4 (7.2), and 15.3 (7.9) nutritional interventions were done. Buddies reported carrying out 23.7 (15.5) out of the 24 possible home visits, leading to an adherence rate of 98.62%. Furthermore, they reported delivering 16.6 (13.4) nutritional and 17.5 (15.9) physical interventions, respectively. No differences were found in the self-perceived reported number of visits or nutritional and physical interventions between buddies and prefrail/frail participants.

### 3.6. Continuation after 12 Months

For this analysis, participants were interviewed by telephone. The home visits were regularly performed by 4.2%, sporadically by 25% of the participants, and 70% did not answer that question. The mean number of home visits in the last 6 months was 19.3 (15.3), which resulted in a mean number of 18.0 (16.3) physical training sessions and in 13.75 (15.05) nutritional interventions.

## 4. Discussion

To sum up the results, our study showed high adherence rates regarding the execution of the home-visits, but showed significant differences in the realization of the actually delivered intervention compared to the study protocol. So, we conclude that adherence rate alone is not adequate to evaluate the implementation of an intervention. Moreover, “being there” is the prerequisite for measures—but cannot show what was done during the intervention, i.e., it does not give any information on the quality of the intervention. Thus, comprehensive documentation led to an important gain in knowledge on how an intervention was done—and was accepted by the participants. These findings led to important questions:

– How much of the planned intervention has to be done to gain effects?
– How should the overall adherence to an intervention respectively the performed quality be described?
– Is a psycho-social context even more important than a physiological context?

The optimum weekly balance of physical activity described in international and national guidelines would be at least two times per week performing muscle strengthening training and additionally at least 150 min of moderate to vigorous intense aerobic physical activity for both, healthy individuals and those suffering from chronic conditions or diseases [41]. The actual challenge is not to postulate only the optimum of the weekly amount of physical activity, the public health challenge is to find feasible interventions leading to long-term effects on lifestyle (changes). Thus, physiological criteria must be used to enable the measuring of effects, but especially in frail and generally in people with low physical function capacities, thresholds to gain effects are low. What is effective has already been known for decades [42], but achieving long-term sustainability is not only based on effectiveness.

The results of our study emphasize that a monitoring system or, as a minimum, a comprehensive documentation of social interaction/support would be helpful to explain adherence to an intervention and consequently study outcomes. For patients who need support to change their personal lifestyle, social interaction is a key factor in achieving lifestyle goals. Thus, social support should not be seen as a “nice to have” in an intervention plan—it should be an integral part of lifestyle management.

Regarding the traditional adherence parameter, our study showed an adherence rate of 72.5% in the social support (SoSu) group and 80.5% in the physical activity and nutritional intervention (PTN) group after 24 weeks for the home-based physical activity and nutritional intervention carried out by lay volunteers (“buddies”) for community-dwelling prefrail/frail older adults. Our adherence rate was comparable to previously reported research where the programs were carried out by health-care professionals [20]. Another published home-based program carried out by health professionals showed an adherence rate of 66% [23], while Vredde et al. reported an attendance of 90% in exercise classes [43]. In a paper published by Stineman et al., the program consisted of a 2-week community-based initial phase followed by a 12-week home-based maintenance phase [44]. Only 1 of 87 participants reported exercising at home after 12 weeks. In a study by Vestergaard et al., a home-based video exercise intervention for community-dwelling older frail women was implemented and evaluated [45]. Within this study, participants exercised for 25 min, three times a week. After 5 months, 17% of the participants dropped out. From the retained sample, the adherence to the protocol was 89.2%. The quality of the performed exercises was not measured, but it was found that clear instructions and supervision were important in achieving the best possible adherence: after an initial 2-week instruction phase, the majority of the participants did not perform home exercises correctly when left on their own [46]. Additionally, Niemela et al. reported that exercise programs had become a regular home-based habit for 88.5% of their subjects [19]. When compared to community-based exercise programs, Aartholathti et al. reported an adherence rate of 55% in a home-based program [18]. In our study, 59.5% of the PTN group realized ≥75% of the offered home visits. Spink et al. reported that, in a home-based program, 52% of the participants completed ≥75% of the three requested exercise sessions per week [23]. Barker et al. reported that, in the retained sample, 95% of the participants attended ≥75% of the offered classes [24]. Interpersonal contact has been found to be a very important factor for prefrail/frail persons to participate in lifestyle programs [11].

Within this study, a comprehensive documentation was used to determine the quality of the delivered intervention. The number of sets performed during the home visits was a key parameter for the exercise regime. The de facto realized number of sets per visit was 1.21 ± 0.58, although 2 sets were postulated in the study protocol. This is admittedly less than planned and presents an important finding in itself. After talking to the buddies, they indicated that it was not possible to perform more sets as this was found to be the maximum tolerated by both the prefrail/frail older persons and the buddies within the 60-min time period, given that, within this time period, both social contact and the nutritional and physical interventions were supposed to be carried out. It should, however, be noted that social contact is extremely important for prefrail/frail older persons. Therefore, the social interaction part needs to be considered as an equally important part of an intervention in future research involving prefrail/frail older persons. In the conception of this study, this prerequisite was underestimated when considering the time plan, and thus the targeted number of sets that was described in the study protocol was not reached. If the number of sets was to be increased, the education of the buddies would need to be expanded and a time-management module integrated. However, there is some literature reporting that a one-set regime is also effective, especially in people with low muscle strength [47]. To determine the overall weekly training workload, the number of performed sets is just one of several parameters. Thus, the weekly performed number of exercise sessions, the number of different exercises, the number of repetitions of sets/exercises, and the workload/repetition must be known to precisely describe the overall weekly workload [48]. According to our findings on muscle strength, we suppose that both regimes were effective because in very weak individuals regular exercising is the most important issue [49,50]. Titze et al. evaluated in a sport-club-based exercise program for people with risk factors for non-communicable diseases, that was supervised from local lay-instructors and not from exercise professionals, that ¾ of the instructors fulfilled the framework of the standards—with several differences in the actual implementation [51,52]. Effects on physical activity were independent of these differences.

A major strength of this study is the fact that it was carried out in a community setting. Furthermore, the fact that the lay volunteers achieved a good adherence rate, which was comparable to interventions carried out by health professionals, presents a novel result within this field of research. The assessment of adherence with a log book which was filled in immediately after each visit is another strength. Comparing visit-by-visit documented adherence differs greatly from self-perceived adherence assessed at only one point in time for a certain period. Furthermore, based on the comprehensive documentation, the data that were found in this study help interpret the outcomes from an interventional study that were published earlier [49,53]. This potential should be used in further health-enhancing strategies in prefrail/frail community-dwelling older adults. Another strength is that continuation was assessed six months after study interventions were terminated. This strength is linked to a limitation, because only one-third of all buddies contributed data to this analysis. It is a well-known challenge in lifestyle interventions to minimize the number of lost participants to long-term follow-up. The small number of participants who provided data for the 12 months follow-up is a limitation. Another limitation of the study is that it was not possible to evaluate the level of the nutritional intervention in detail. Similar to the nutritional aspects, social support was not documented, because the study team was focused on organizational aspects and parameters to concretize exercise training. Thus, an adequate section in the log book should be added for further research.

## 5. Conclusions

In conclusion, lay volunteers can carry out home-based lifestyle interventions in community-dwelling older people, resulting in good adherence rates. Further parameters to document the intervention help explain adherence rates—as well as other study outcomes. Comprehensive documentation is needed in lifestyle interventions: it should not be focused only on the intervention itself but also on the level of social support, because the interpersonal contact is a decisive factor for the successful implementation of lifestyle programs. Within the current study, it was shown that laymen can efficiently carry out a physical activity and nutritional support program in community dwelling older people. However, similar interventions can easily be transferred to other settings like community senior living centers that would also benefit from a program like this.

## Figures and Tables

**Table 1:** Baseline characteristics of the participants presented as mean, standard deviation, or frequencies.

	Prefrail/Frail Participants (n = 80)	Buddies (n = 70)
Age (years)	82.6 ± 8.1	60.1 ± 6.9
Sex (Female, %)	83.8	89.1
Body height (cm)	162.1 ± 8.6	167.1 ± 6.9
Body weight (kg)	71.4 ± 12.7	71.3 ± 14.9
Body mass index (kg/m^2^)	27.4 ± 4.4	25.6 ± 5.4
Waist circumference (cm)	103.5 ± 11.5	93.3 ± 14.8
Hand grip strength (kg)	16.9 ± 7.3	32.1 ± 7.9
Share-FI categories (%) *		
Robust	1.3	
Prefrail	35.0	
Frail	63.7	
Educational level (%)		
Primary	52.5	21.4
Secondary	35.0	54.3
Tertiary	12.5	18.6
Missing		05.7
Living with a partner (%)	17.5	55.9

* SHARE-FI Categories were only evaluated in the prefrail/frail participants and not in their buddies.

**Table 2:** Adherence parameters of prefrail and frail participants as mean, standard deviation, and frequencies.

	PTN		SoSu	
	Weeks 1–12	Weeks 13–24	Weeks 1–12	Weeks 13–24
Number of weeks between follow-ups	10.5 ± 2.8	9.9 ± 2.3 ^1^	9.4 ± 2.6	10.8 ± 2.1 ^3^
Number of visits per week	1.6 ± 0.4	1.6 ± 0.7 ^2^	1.5 ± 0.5	1.5 ± 0.5 ^4^
Adherence rate	84.0%	80.5%	75.5%	72.5%
Adherence category ^5,6^				
High adherent (%)	61.9	59.5	47.4	36.8
Moderately adherent (%)	26.2	14.3	21.1	15.8
Low adherent (%)	2.4	7.1	5.3	7.9
Very low adherent (%)	0	2.4	5.3	2.6
Drop outs (% of the whole sample)	11.9	16.6	23.7	36.8

^1^ Paired samples *t*-test follow-up 1&2; *p* = 0.469. ^2^ Paired samples *t*-test follow-up 1&2; *p* = 0.880. ^3^ Paired samples *t*-test follow-up 1&2; *p* = 0.612. ^4^ Paired samples *t*-test follow-up 1&2; *p* = 0.493. ^5^ Chi-Square test PTN group follow-up 1&2: *p* = 0.018, df = 9, X2 = 19.96. ^6^ Chi-Square test SoSu group follow-up 1&2: *p* = 0.366, df = 3, X2 = 3.17. PTN = physical training and nutrition group; SoSu = social support group.

**Table 3:** The dose of effectively delivered interventions in visit 2 and 3, presented as mean ± standard deviation and frequencies (%).

	Planned Intervention	Actual Intervention	Dose (%)	Actual Intervention	Dose (%)	*p* *
		after 12 weeks *		after 24 weeks **		
Home visit per week (times)	2	1.6 ± 0.5	80	1.6 ± 0.6	77	0.562
Duration of home visit (hours)	1	1.4 ± 0.5	140	1.5 ± 0.8	154	0.259
Intervention missed due to prefrail/frail person	0	4.4 ± 4.5		4.5 ± 4.9		0.880
Intervention missed due to buddy	0	2.2 ± 3.1		2.0 ± 2.3		0.628
Number of physical training intervention per home visit	1	0.4 ± 0.5	41	0.8 ± 0.6	81	<0.001
Number of circles per home visit	2	2.1 ± 0.7	107	1.2 ± 0.6	60	0.006
Number of conducted strength exercises per home visit	6	6.4 ± 5.5	106	5.3 ± 1.3	89	0.016
Number of repetitions per exercise	15	7.3 ± 6.5	48	13.1 ± 4.6	87	<0.001
Number of nutritional interventions per home visit	1	0.6 ± 0.7	56	0.5 ± 0.3	47	0.168
Number of “Healthy for life plate” interventions per home visit	1	5.2 ± 6.7	521	0.2 ± 0.3	22	
Number of nutritional messages delivered per home visit	1	5.1 ± 7.6	507	0.3 ± 0.4	26	

* *t*-test for independent samples. After 12 weeks: only patients from the PTN were included in the analysis. ** After 24 weeks: as the SoSu group received the same intervention as the PTN group, both groups were analyzed together.

**Table 4:** Chi-square test for variables associated with adherence in the prefrail/frail participants.

		High Adherent	Moderate, Low and Very Low Adherent	*p*
Age	Younger than 81	9 (23.1%)	12 (60.0%)	0.006
	Older than 81	30 (76.9%)	8 (40.0%)	
Sex	Female	35 (89.7%)	16 (80.0%)	0.258
	Male	4 (10.3%)	4 (20.0%)	
Education level	Primary	24 (61.5%)	8 (40.0%)	0.124
	Secondary	13 (33.3%)	8 (40.0%)	
	Tertiary	2 (5.1%)	4 (20.0%)	
SHARE-FI	Robust	2 (5.1%)	4 (20.0%)	0.038
	Prefrail	22 (56.4%)	5 (25.0%)	
	Frail	15 (38.5%)	11 (55.0%)	

**Table 5:** Chi-square test for variables associated with adherence in buddies.

		High Adherent	Moderate, Low and Very Low Adherent	*p*
Age	Younger than 59	15 (60.0%)	6 (40.0%)	0.220
	Older than 59	10 (40.0%)	9 (60.0%)	
Sex	Female	27 (93.1%)	13 (86.7%)	0.481
	Male	2 (6.9%)	2 (13.3%)	
Educational level	Primary	7 (25.0%)	4 (28.6%)	0.853
	Secondary	15 (53.6%)	8 (57.1%)	
	Tertiary	6 (21.4%)	2 (14.3%)	
